# Mortality among hospitalized COVID-19 patients in the West Cameroon Regional Hospital

**DOI:** 10.11604/pamj.2022.41.122.32349

**Published:** 2022-02-11

**Authors:** Ketina Hirma Tchio-Nighie, Iliasou Njoudap Mfopou, Francois Nguegoue Tchokouaha, Jerome Ateudjieu

**Affiliations:** 1 Department of Health Research, Meilleur Accès aux Soins de Santé (M.A. Santé), Yaoundé, Cameroon,; 2 Department of Public Health, Faculty of Medicine and Pharmaceutical Sciences, University of Dschang, Dschang, Cameroon,; 3 Department of COVID-19 Management, Bafoussam Regional Hospital, Bafoussam, Cameroon,; 4 Department of Internal Medicine, Bafoussam Regional Hospital Center, Bafoussam, Cameroon,; 5 Division of Health Operations Research, Ministry of Public Health, Yaounde, Cameroon

**Keywords:** COVID-19, in-hospital lethality, fatal outcome, Africa, Cameroon

## Abstract

**Introduction:**

despite its relatively low case-fatality rate, COVID-19 is a concern with high mortality and morbidity of hospitalized cases. This study was conducted to assess the relationship between time to consultation, presence of respiratory complications at hospital admission and fatal outcome of COVID-19 cases.

**Methods:**

this was a case control study with data collected from records of all patients admitted in the Bafoussam Regional Hospital (BRH) from March 2020 to April 2021. Cases were patients with a fatal outcome and controls were patients that were discharged. The association between the delay in seeking care, dyspnea and blood oxygen level at admission, and fatal outcome was assessed by estimating crude and adjusted odd ratio.

**Results:**

of 400 included patients, 239 (59.75%) were male, 84 (23.73%) health professionals and 144 (36.0%) aged 64 years and above. On admission, 236 patients presented at least one sign of respiratory complication. The mean duration of hospitalization was 11.4 days and 120 (30.0%) admitted patients died. Seeking care before the end of the first day of symptom onset (adjusted (A) OR=0.44 [0.21-0.97]) or within the first three days (AOR=0.48 [0.26-0.89]) significantly reduced the risk of fatal outcome, whereas waiting seven days (AOR=0.74 [0.42-1.33]) did not change this risk. Presenting dyspnea (AOR=2.39 [1.32-4.31]) or blood oxygen level <95% (AOR=3.67 [1.37-9.83]) significantly increased the risk of fatal outcome.

**Conclusion:**

mortality was one in three patients. Early arrival at the hospital helped to reduce the risk of mortality unlike presenting respiratory complication that increased the risk. Health interventions contributing for early detection and link of COVID-19 cases to care before respiratory complications occur are expected to reduce mortality in COVID-19 patients.

## Introduction

The control of COVID-19 like many other epidemics included interventions to reduce its propagation, mortality and its burden on health systems and socioeconomic environments. Despite the various control measures attempted, COVID-19 is still characterized by a high morbidity with more than 178 million positive cases reported worldwide as of June 21, 2021 [1]. Associated to this high morbidity are reported relatively low case-fatality rates of 2.1% worldwide, 2.7% in Africa and 1.6% in Cameroon at the same date [1, 2]. At health facilities level, studies have reported mortality that varied from 15% to 47.6% according to the study sites and the type of patients managed [3-7]. Unlike the determinants associated with morbidity which are known, the determinants of these high in-hospital mortality are not known. Estimating this mortality and its determinants should help generate information that can guide the choice of interventions to be implemented to contribute to their reduction.

COVID-19 is characterized by a high morbidity rate, with a proportion of severe cases in a category of patients, including elderly patients and those with risky comorbidities; and also, by a rapid and usually fatal course of these severe cases [8]. The pathophysiology of the disease characterized by diffuse alveolar damage following invasion of the upper and lower airways with systemic consequences would explain mortality in those at risk [9-11]. Review of data in the United States of America reveals a higher mortality of COVID-19 on weekends, raising the question of whether delays in seeking care may have any relationship to risk of death [12]. It would be plausible that prolonged delays between symptom onset and formal consultation would delay the provision of palliative care that could limit the occurrence of potentially lethal complications of the disease. Prompt management of these cases would be necessary to prevent the occurrence of complications and deaths resulting from COVID-19. Early detection, diagnosis, isolation, and clinical management of patients would help reduce morbidity and mortality [8]. The health system in low- and middle-income countries, including Cameroon, is characterized by a relatively limited technical platform and access to care, which may contribute to an increased risk of mortality in the event of an epidemic [8, 13, 14]. This situation has been described in the context of the control of other diseases with epidemic potential in Cameroon [13, 15].

In Cameroon, the epidemic began in March 2020. The response included governmental measures and the organization of the curative care system including the establishment of specialized centers for the management of COVID-19 cases [16, 17]. A review of the different national situation reports (sitrep) shows that the mortality of the disease does not vary significantly from one region to another and from one epidemiological week to another [18-22]. The distribution of deaths is reported in these sitreps but do not allow to identify the hospital share of these deaths [21].

Although the literature has identified groups at high risk of mortality, there is still a need for information on interventions to reduce in-hospital mortality [3-7]. To the best of our knowledge, no study has been published in Cameroon describing the distribution of in-hospital mortality scores and the determinants related to this mortality. Such study is expected to generate information that can guide the choice of interventions to be implemented to contribute to the proper management of COVID-19 and reduction of the mortality of COVID-19. The present study is proposed to describe the clinical state of COVID-19 cases and identify factors that may be associated to the fatal outcome of these cases by responding to the questions: does seeking care in health facility within the first day, 3 days, 7 days after onset of the first perceived symptoms of COVID-19 increase risk of death among hospitalized COVID-19 cases? And is the risk of death higher in patients consulting with any respiratory complication sign compared to those with no respiratory complication sign?

## Methods

**Study design:** this was a case control study that targeted all COVID-19 patients admitted in the Bafoussam Regional Hospital (BRH) from March 2020 to April 2021. Data were collected from individual patients´ medical records. Cases were patients with a fatal outcome and were matched to controls (patients that were discharged) with respect to age groups. The association between the delay in seeking care, dyspnea and blood oxygen level at admission, and fatal outcome was assessed by estimating crude and adjusted odd ratio (OR).

**Study site and period:** the study was conducted at the Bafoussam Regional Hospital which is the reference hospital in the Western Region of Cameroon. It was selected as the reference center in charge of the management of symptomatic COVID-19 patients in the West Region of Cameroon. Data were collected from January to April 2021.

**Study population:** were eligible all hospitalized COVID-19 cases that were confirmed either by a reverse transcriptase polymerase chain reaction (RT-PCR) or a rapid diagnosis test (RDT). Cases were patients whose outcome was death and controls those who were discharged. Patients who did not have a documented outcome or information on the exposures were excluded. One case was matched to one control with respect to the age group.

**Sample size:** the sample size was estimated at 407 patients assuming: a study power of 80%, that the risk of death will be about 50%, and an expected odd ratio of at least 2 between exposures and death. The estimate was guided by the WHO manual for sample size determination in health practices [23].

**Data collection:** data were collected using an extraction grid that was developed by the study team and pre-tested in a district hospital in the West Region of Cameroon. Key variables collected were the delay in seeking care following symptom onset, signs and symptoms at hospital arrival, symptoms during hospitalization and outcome at the end of hospitalization.

**Data collection procedures and management:** data were extracted from individual patient by patient hard copies medical records and recorded in a Microsoft Excel collection grid. Missing data were confronted to an existing database in the hospital. The data collected was cross-checked by a second person and treated at a weekly basis to detect outliers.

**Data analysis:** for descriptive analyses, proportions and means were estimated. Crude and adjusted odd ratios were used to estimate the associations between the delay in seeking care, dyspnea and blood oxygen level at admission, and fatal outcome. Adjustments were done with sex, occupation, health district, presence of at least a comorbidity. P values <0.05 were considered significant. All analysis were done using Stata 16 and Epi Info Software.

**Ethical consideration:** the present study was expected to produce information to identify intervention needs and targets to reduce in-hospital mortality. The research protocol including the objectives and study procedures was presented to the authorities of the BRH and the head of the COVID-19 care department prior to obtaining the implementation agreement and access to the patients' data. No personnel information on patients were collected. The research protocol of the present study was approved by the institutional ethics committee of the BRH with reference N°11645/L/MINSANTE/SG/DRSPO/HRB/D.

## Results

**Figure 1:**
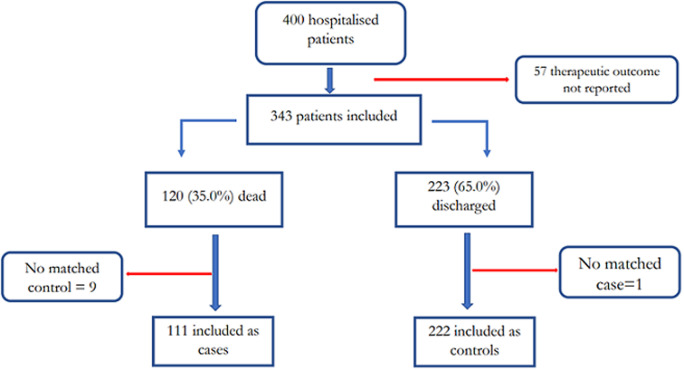
consort flow of the study “clinical status of consulting COVID-19 patients in a Cameroon regional hospital”

**Participants:** between March 1, 2020 and April 5, 2021, 400 COVID-19 cases were hospitalized at the Bafoussam Regional Hospital. Of these 400, 343 (85.8%) fulfilled the inclusion criteria, 111 patients were included as cases and 222 as controls. Figure 1 presents the number of patients reached and included in each stage of the study.

**Participants´ characteristics:** the average age was 55.8 years and 144 (36.0%) were aged 64 years and above. A total of 239 (59.75%) were men and 84 (23.73%) were health professionals. Table 1 presents detailed participants´ characteristics.

**Table 1:** participants’ characteristics

	Frequency	Proportion (%)
**Age (years)**		
≤14	1	0.3
15-49	129	32.3
50-64	126	31.5
>64	144	36.0
**Profession**		
Health professionals	84	21.0
Housewife	61	15.3
Self employed	50	12.5
Employee	44	11.0
Retired	38	9.5
Civil servant	33	8.3
Farmer	17	4.3
Student	9	2.3
Religious	6	1.5
Unemployed	3	0.8
Undocumented	46	11.5
**Marital status**		
Maried	337	84.3
Single	45	11.3
Widow	5	1.3
Undocumented	13	3.3

**Figure 2:**
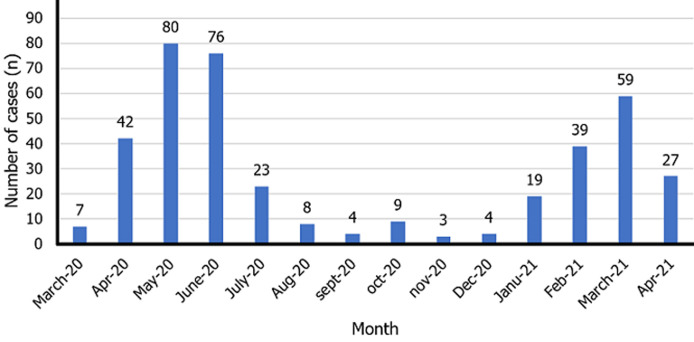
temporal distribution of COVID-19 cases managed at the Bafoussam Regional Hospital

**Distribution of COVID-19 confirmed cases:** Figure 2 presents the monthly distribution of COVID-19 positive cases managed at the Bafoussam Regional Hospital. Two peaks of absolute increase in cases are noted, one from April to July 2020 and the other from January to April 2021.

**Figure 3:**
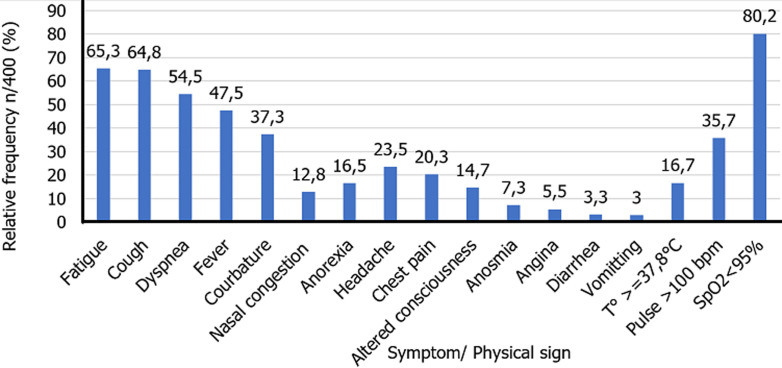
symptomatology and parameters of COVID-19 cases managed at the Bafoussam Regional Hospital

**Symptomatology and parameters at patient admission:** the symptomatology and physical parameters at admission of COVID-19 positive cases received at the BRH from March 2020 to April 2021 are presented in Figure 3. The two most frequent symptoms were fatigue (65.3%), cough (64.8%) and 218 (54.4%) had signs of respiratory complication. The least frequent symptoms were digestive (diarrhea (3.3%) and vomiting (3.0%)). More than 80% patients had blood oxygen level <95%.

**Comparison of sociodemographic and clinical characteristics of cases and controls:** Table 2 presents the comparisons of sociodemographic and clinical characteristics of the cases and controls. There was no difference in distribution with regard to age and presence of hypertension.

**Table 2:** description and comparison of cases and controls

Characteristics	Cases n (%)	Controls n (%)	Chi 2	P-value
**Sex**				
Male	71 (64.0)	120 (54.0)	2.97	0.09
**Age**				
≤64 ans	62 (55.9)	57 (25.7)	29.35	<0.001
**District**				
Mifi	54 (52.4)	136 (64.8)	4.41	0.04
**Profession**				
Health professional	14 (14.6)	67 (33.3)	11.52	<0.001
**At least 1 comorbidity**				
Yes	85 (78.0)	124 (59.3)	11.06	<0.001
**Presence of diabetes**				
Yes	49 (57.7)	52 (41.9)	4.99	0.03
**Presence of hypertension**				
Yes	49 (57.7)	65 (52.4)	0.56	0.47

**Association between time to consultation and fatal outcome:** the mean time from first symptom onset to admission was 7.2 days. A total of 101 (25.3%) patients consulted on the first day, 145 (36.3%) during the first three days, 266 (66.5%) during the first 7 days and 122 (30.5%) after the seventh day following the onset of the first COVID-19 symptoms. Table 3 presents the association between time to consultation and fatal outcome. Seeking care during the first 24 hours or the first three days significantly reduced the risk of death, whereas there was no association between seeking care during the first seven days of symptomatology and the occurrence of death. These relationships persisted when estimating the crude OR and adjusted OR taking into account sex, the profession, the health district, the presence of at least one comorbidity as potential confounders.

**Table 3:** association between time to consultation and fatal outcome

	Crude OR			Adjusted OR		
	OR	CI at 95%	P-value	OR	CI at 95%	P-value
≤ 1 day	0.38	[0.21; 0.70]	0.002	0.44	[0.21; 0.97]	0.01
≤ 3 days	0.50	[0.30; 0.83]	0.006	0.48	[0.26; 0.89]	0.01
≤ 7 days	0.90	[0.55; 1.47]	0.68	0.74	[0.42; 1.33]	0.32

* CI: confidence interval

**Association between presence of respiratory complications at hospital admission and fatal outcome:** the presence of dyspnea or blood oxygen level less than 95% or both at admission was significantly associated with an increased risk of death. The trend persisted with respect to the crude and adjusted OR values taking into account as confounders the sex, occupation, health district, presence of at least one comorbidity. The association between the presence of dyspnea or blood oxygen level less than 95% at entry and fatal outcome is presented in Table 4.

**Table 4:** association between the presence of dyspnea or blood oxygen level less than 95% at entry and fatal outcome

	Crude OR			Adjusted OR		
	OR	CI at 95%	P-value	OR	CI at 95%	P-value
Dyspnea	2.88	[1.75; 4.74]	<0.001	2.39	[1.32; 4.31]	0.004
Blood oxygen saturation (SpO2) <95%	3.69	[1.54; 8.83]	0.003	3.67	[1.37; 9.83]	0.009
Dyspnea and SpO2<95%	3.36	[2.02; 5.58]	<0.001	2.82	[1.54; 5.15]	<0.001

* CI: confidence interval

## Discussion

This study was conducted to describe the clinical status of cases at hospital entry and factors that may influence the risk of death in COVID-19 patients. A total of 145 (36.3%) patients presented to the hospital within the first three days after the onset of the first symptoms and 122 (30.5%) after the seventh day. At entry, 218 (54.5%) and 202 (80.2%) patients had dyspnea and blood oxygen desaturation, respectively. Of the 236 patients who had at least one respiratory complication sign, 136 (57.6%) were able to receive oxygen. Anticoagulants were prescribed in 117 (81.3%) of the patients with an age greater than 64 years, 101 (76.5%) who had hypertension, and 95 (79.2%) who had diabetes. Of the 343 patients with known therapeutic outcomes, 223 (65.0%) were discharged and 120 (35.0%) died. Seeking care within the first day (ORA=0.44 [0.21-0.97]) or within the first 3 days (ORA=0.48 [0.26-0.89]) following symptom onset significantly reduced the risk of death. The presence of dyspnea (ORA=2.39 [1.32-4.31]) or blood oxygen saturation <95% (ORA=3.67 [1.37-9.83]) significantly increased the risk of death.

Early diagnosis and timely initiation of treatment play a critical role on disease prognosis [24]. The time to seek care after disease onset is an important parameter in assessing access to care and a determinant of treatment outcome. Early care-seeking is recommended to prevent the development of complications and the risk of mortality, but to the best of our knowledge, there are no studies documenting appropriate time frames for COVID-19 [25]. This lack of information explains why this issue was investigated in the present study. The present study reveals a delay in seeking care after the onset of first symptoms and consultation ranging from 0.0 days to 45.0 days with a mean of 7.2 days. Despite a difference in time period and target population, other studies conducted in France and China have found a similar mean duration of approximately 7 days [3,5,26]. Data were not collected to investigate reasons for the choice of delays to seeking care after the onset of symptoms, as in other published studies. There are several reasons that may contribute to the choice of these delays. These may include lack of knowledge or misinterpretation of the symptomatology in the initial phase of the disease, inappropriate choice of therapeutic route, limited access to screening, limited access to information, geographic inaccessibility, or fear related to transmission or stigma of the disease [27,28]. Longer delays in seeking care could imply more people exposed to the disease and delayed management of some complications. Studies should be conducted to explain and anticipate longer delays in seeking care for suspected cases.

In the context of several diseases, particularly communicable diseases, mortality is a parameter for evaluating the response to epidemics. It is dependent on the therapeutic course of patients before and during hospitalization [15]. In the context of other epidemic prone diseases, the frequency of mortality has been distributed in three groups; mortality at home, on the way to the hospital and in the hospital [15]. The present study investigated hospital mortality in patients managed for COVID-19 and found that one in three hospitalized patients died. A number of studies documenting this mortality have been conducted in China and France and have shown similar mortality rates of 32.5%, 47.6% and 19% [4, 6, 7]. This similarity despite the differences in context and epidemic period raises the fact that therapeutic protocols and technical platforms may not have a significant impact on mortality at the current state of knowledge. The data from our study would have made it possible, if the sample size was sufficient, to explore the contribution of access to recommended protocols such as the use of oxygen in patients with signs of respiratory complication, which was 57.6% in the present study, and the use of anticoagulants in patients at risk of thrombosis, which was 77.2% for patients with diabetes and 74.8% for patients with hypertension [25,29-31]. If the coverage of patients in need of these protocols has been limited, there is a need for knowledge about their effectiveness in preventing these deaths.

COVID-19 is associated with respiratory and multi-systemic inflammatory syndrome including cardiovascular, renal, pulmonary, hematological, gastrointestinal, dermatological and neurological involvement [9, 31-33]. Studies have shown that early management of a number of COVID-19-related syndrome are determinants of the development of complications [30]. The results of the present study show that delaying seeking care until the end of the first day or within the first three days after the onset of COVID-19 symptoms significantly reduced the risk of death, whereas waiting up to 7 days did no longer influence this risk. To the best of our knowledge, no study has investigated the association between time to seek care and fatal outcome. This association documented in the present study should be confirmed by other studies and could be explained either by the delay in prevention of certain lethal syndrome or by the inadequacy of the technical platform to respond to the complicated phases of manifestation of the disease.

COVID-19 is associated with infection of epithelial and immune cells of the respiratory tract and infection of pneumocytes that express angiotensin converting enzyme 2 (ACE2) and can cause an inflammatory response that generates the cytokine storm phenomenon that results in respiratory distress of varying severity, which in its most severe form can lead to acute respiratory distress syndrome [31]. SARS-CoV-2 also destroys alveolar cells, which are essential for the exchange of oxygen and carbon dioxide, which would explain low blood oxygen saturation levels in patients with COVID-19 [34]. These manifestations, which may result in death by physiological asphyxia, require specialized management. The present study found that the presence of dyspnea or blood oxygen saturation of less than 95% or both on admission was significantly associated with an increased risk of death. The current literature available to us has not explored this issue with the same methodology. Plausible explanations for this association could include the inadequacy of the technical platform, the clinical condition of the patients, the inadequacy of the proposed protocol and the failure to comply with national guidelines in terms of detection and monitoring of suspected cases in the community on one hand, and the insufficient use of oximeters for monitoring cases in the community on the other.

The interpretation and use of these results should take into account a number of limitations, including poor data completeness in some patients, lack of therapeutic outcome in some patients (especially referred and escaped patients), memory bias on the dates of onset of symptoms, and non-standardization of recording and measurement methods. This could contribute to the over- or under-estimation of some indicators and could alter the meaning and interpretation of some estimated associations. The number of participants for did not meet the calculated sample size; this could increase the fluctuation interval around the estimated associations. Also, non-generalizability of findings could be considered as a limitation as this study was conducted in only one management COVID-19 site that do not necessarily reflect other COVID-19 management centers and their patients.

## Conclusion

Patients consult on average 7.2 days after the onset of symptoms. On admission, about 3/5 patients have at least one sign of respiratory complication. Almost one third of hospitalized patients had a fatal outcome. Seeking care on the first day or within the first three days after the onset of symptoms significantly reduces the risk of death. Presenting a sign of respiratory complication including dyspnea or blood oxygen desaturation significantly increased the risk of death. Following the findings of the present study, we recommend to health authorities to establish a mechanism to ensure the use of national guidelines for the management of COVID-19, design procedures to guide the dealing with suspected symptoms to be made available to communities, establish mechanisms to improve early case detection and early prevention of complications, and establish a communication plan to reduce delays in seeking care for suspected cases. To researchers, we recommend to investigate and address the major determinants of mortality related to COVID-19, identify the most efficient therapeutic protocols for the management of respiratory complications for health facilities with limited technical resources, identify community-based interventions that would reduce the time to detect suspected cases and anticipate their complications, assess the adequacy of the proposed technical facilities and the needs for the management of complicated cases of COVID-19, and verify the consistency of the findings of the present study by prospective studies with data collection from hospitalized patients.

### What is known about this topic

- *Determinants of COVID-19 transmission are known;*
- *COVID-19 is associated with respiratory and multi-systemic inflammatory syndrome*.

### What this study adds

- *This is one of the first studies investigating the determinants of in-hospital mortality;*
- *This study reveals that seeking care between the first and the third day following COVID-19 symptom onset reduces significantly the risk of death;*
- *This study reveals that more than 80% of COVID-19 patients reach the hospital with an oxygen blood saturation level less than 95%*.
